# Association between Physical Activity Guidelines and Sedentary Time with Workers’ Health-Related Quality of Life in a Spanish Multinational Company

**DOI:** 10.3390/ijerph19116592

**Published:** 2022-05-28

**Authors:** Paola Gómez-Redondo, Victoria Marín, Javier Leal-Martín, Carlos Ruiz-Moreno, Verónica Giráldez-Costas, Pilar Urdiola, Ignacio Ara, Asier Mañas

**Affiliations:** 1 GENUD Toledo Research Group, University of Castilla-La Mancha, 45004 Toledo, Spain; paola.gomez@uclm.es (P.G.-R.); javier.leal@uclm.es (J.L.-M.); ignacio.ara@uclm.es (I.A.); 2 CIBER of Frailty and Healthy Aging (CIBERFES), 28029 Madrid, Spain; 3 Media and People Services Department, Health Area, Grupo Red Eléctrica de España SAU, 28109 Madrid, Spain; victoria.marin@ree.es (V.M.); purdiola@ree.es (P.U.); 4 Exercise Physiology Laboratory, Camilo José Cela University, 28692 Madrid, Spain; cruizm@ucjc.edu (C.R.-M.); vgiraldez@ucjc.edu (V.G.-C.); 5 Center UCM-ISCIII for Human Evolution and Behavior, 28029 Madrid, Spain; 6 Faculty of Education, Complutense University of Madrid, 28040 Madrid, Spain

**Keywords:** workplace, exercise, well-being, office, mental health

## Abstract

Workers spend a large amount of time working, limiting the possibility of meeting physical activity (PA) guidelines. A better health-related quality of life (HRQoL) provides benefits for the employee and company. The aim of this study was to analyse the associations of four behavioural categories between compliance with PA guidelines (aerobic and strength training) and sedentary time with workers’ HRQoL. We classified the sample into four categories: (1) “Physically active & low sedentary”, (2) “Physically active & high sedentary”, (3) “Physically inactive & low sedentary”, and (4) “Physically inactive & high sedentary”. Student’s *t*-tests for two independent samples and a multiple linear regression adjusted for covariates were performed. A total of 1004 employees of the multinational company Grupo Red Eléctrica participated. Compliance with PA guidelines and a low level of sedentarism were associated with higher HRQoL (*p* < 0.001). Compared to “physically inactive & high sedentary”, “physically active & low sedentary” and “physically active & high sedentary” workers obtained a better HRQoL (B = 5.47; *p* = 0.006 and B = 4.10; *p* = 0.003; respectively). In this sample of Spanish workers, being physically active was associated with a better HRQoL, even in those with high sedentary time. Experimental studies are needed to confirm our results.

## 1. Introduction

Full-time workers typically spend approximately one-third of their day or more in the workplace [1], which is generally associated with more time sitting and less time spent walking or standing when compared to non-working days [2]. Sedentary behaviours are common during working hours, and sometimes, workers do not compensate for excessive sedentary behaviour at work by reducing sedentary behaviour during leisure time [3]. Current World Health Organization (WHO) guidelines for adults involve weekly volume of aerobic activities of at least 150 min of moderate-intensity physical activity (PA), or 75 min of vigorous-intensity PA, or an equivalent combination of moderate to vigorous PA (MVPA), as well as regular muscle-strengthening activities at least two days per week (e.g., strength exercises such as weight lifting) [4]. People working in jobs characterized by long periods of sedentary work have limited time to meet PA guidelines [1]. In this regard, 60% and 67% of Spaniards do not practice any type of moderate or vigorous PA, respectively [5]. Likewise, a recent survey conducted in Europe shows that 82.7% of the European people did not satisfy muscle-strengthening guidelines [6].

Health-related quality of life (HRQoL) is a subjective and multidimensional concept that takes into account at least physical, psychological and social dimensions [7]. This concept applies to different contexts of life in which work plays an important role [8]. Some studies have shown how PA can modulate HRQoL in workers [9,10,11]. However, we still have a long way to go before fully understanding the interrelationships between PA patterns and time of sedentary lifestyle together with the role they can play in improving employees’ HRQoL. Previous research has shown that some adults may exhibit elevated patterns of sedentary time even when meeting PA guidelines [12]. Nevertheless, we could also find the opposite behaviour pattern. Based on the above, it is possible to distinguish four different behavioural patterns (“physically active and low sedentary”, “physically active and high sedentary”, “physically inactive and low sedentary”, and “physically inactive and high sedentary”). Some of these pattern combinations can produce various harmful effects related to health [13,14,15]. For example, Henson et al. [15] found that, compared to physically inactive and highly sedentary adults, the physically active had less liver, visceral, and total abdominal fat, even when combined with high sedentary time. However, this idea has never been applied with HRQoL as an outcome. Additionally, the previously mentioned studies that established the four behavioural categories used them as a reference to classify as physically active or inactive people who barely complied with the WHO aerobic PA guidelines, leaving out the muscle-strengthening guidelines. Although compliance with muscle strength guidelines has been relegated in these studies, the important benefits this type of training has on health are currently well known [16,17,18]. Moreover, as far as our work is concerned, strength training may be another key factor in improving the HRQoL of workers since it has been shown to have multiple beneficial effects on this variable [19,20].

Appreciating the possible consequences for HRQoL from different combinations of mutually exclusive behavioural categories among workers can be advantageous since companies can design better health interventions, proving to be beneficial both from the employee and employer’s point of view. Based on the above, the following research question was formulated: Is there an association between the four mutually exclusive behavioural categories according to compliance with PA guidelines and sedentary time status with HRQoL in workers? Therefore, the objective of this study was to analyse the relationship between the four categories of behaviour related to compliance with the PA guidelines (aerobic and strength) and sedentary time in the HRQoL of the employees of the Grupo Red Eléctrica. Our hypothesis was that all mutually exclusive behavioural categories would be associated with HRQoL in reference to the “physically inactive and high sedentary” category. Physically active workers would have a higher HRQoL, with this score increasing progressively as we approach the “physically active and low sedentary” behavioural category.

## 2. Materials and Methods

### 2.1. Study Design and Procedure

This was a cross-sectional study using a self-administered online survey sent by e-mail to 1810 employees of the multinational Spanish company Grupo Red Eléctrica. Upon accessing the website, employees completed a survey consisting of 39 questions following a structured format based on previously validated studies [21,22,23,24,25]. Seventeen of the questions in the survey were used in this study to assess information related to age, sex, job position, number of diseases, weekly PA guidelines (aerobic and strength), sedentary time and HRQoL. The online survey was completed anonymously in order to preserve the confidentiality of the participants. Data were collected from 21 May to 4 June 2018.

### 2.2. Measures

#### 2.2.1. Health-Related Quality of Life

The visual analogue scale included in the EuroQol-5D questionnaire was used to assess HRQoL [21]. This scale is an instrument with good validity and excellent reliability [26] and consists of asking participants to indicate their own current health status on a rating scale calibrated from 0 (“worst imaginable health status”) to 100 (“best imaginable health status”).

#### 2.2.2. WHO Physical Activity Guidelines

The MVPA was assessed using the short version of the International Physical Activity Questionnaire (IPAQ-SF), which provides repeatable data and has acceptable measurement properties [22]. This questionnaire measures frequency (days per week) and time (minutes per day) spent in moderate (e.g., carrying light weights, cycling at regular speed, or playing doubles tennis) and vigorous (e.g., running, swimming, or cycling at high speed) aerobic activities performed for at least 10 min at a time over the past 7 days. Aerobic status was classified as “physically active” or “physically inactive” on the basis of whether or not participants met the WHO PA guidelines for adults (16). To do so, they had to meet at least one of the following three premises: (1) perform at least 150 min per week of moderate-intensity PA, (2) accumulate at least 75 min per week of vigorous-intensity PA, or (3) an equivalent combination thereof.

To assess subjects’ participation in muscle-strengthening activities (e.g., free weight lifting, weight machines, resistance bands, etc.), questions from the 2014 European Health Survey in Spain (EESE) were used [23]. Thus, subjects were asked about the frequency of muscle strengthening (days per week) performed during a usual week. Based on the strength training guidelines proposed by the WHO [4], those participants who performed two or more days per week were classified as “physically active”, as opposed to those who weekly did not reach the temporal limit of two days, who were classified as “physically inactive”.

Finally, those subjects who met both the aerobic and muscular strength PA guidelines [4], as recommended by the WHO, were classified as “meeting the guidelines”, and, therefore, as “physically active”, as opposed to those who did not comply with one or both guidelines, being in this case classified as “non-meeting the guidelines”, and, therefore, as “physically inactive”.

#### 2.2.3. Sedentary Time

Measurements related to sedentary time were collected both in the work environment and during leisure and free time through the total number of hours per week spent in sedentary activities. Information on sedentary time in the workplace was obtained using the Occupational Sitting and Physical Activity Questionnaire (OSPAQ) [24], which has acceptable reliability and validity measurement properties [27,28]. Workers were asked the following questions: “How many hours did you work during the past 7 days?” and “How would you describe your typical workday during the past 7 days? Distribute 100 percentage points according to the time spent: (a)% Sitting (b)% Standing (c)% Walking (d)% Tasks with high physical demands”. The number of sedentary hours during working time (WSH) was calculated as follows: (% Sitting * Working hours during the last 7 days)/100. In addition, two modified questions from the AusDiab3 questionnaire [25] about participants’ sitting time were used to estimate the amount of sedentary time occurring outside the workplace, remembering to include transportation, watching TV, computer use, mealtime, and other leisure activities (e.g., reading, watching movies) as sedentary times. The number of sedentary hours during leisure time (LSH) was calculated as follows: (weekday LSH × 5) + (weekend LSH × 2). Both WSH and LSH were summed to determine the estimated time spent in sedentary activities in the previous week.

Because sedentary levels in our sample were expected to be predominantly high [29,30], a conservative approach was adopted based on extracting the behaviour-based sedentary status of our population, an approach used in previous studies [13,14]. The total number of sedentary hours per week was used to divide the sample into four quartiles establishing a time of 44 h per week of sedentary activities based on a separation between quartile 1 and the remaining quartiles. In this study, participants residing in the first quartile were classified as “low sedentary”, comprising those workers who spent less than 44 h per week in sedentary activities. The remaining individuals (i.e., those located in quartiles 2, 3, and 4 of sedentary time) who responded performing 44 h or more of sedentary time during the week were classified as “high sedentary”.

#### 2.2.4. Determination of Physical Behaviour Categories

**Figure 1:**
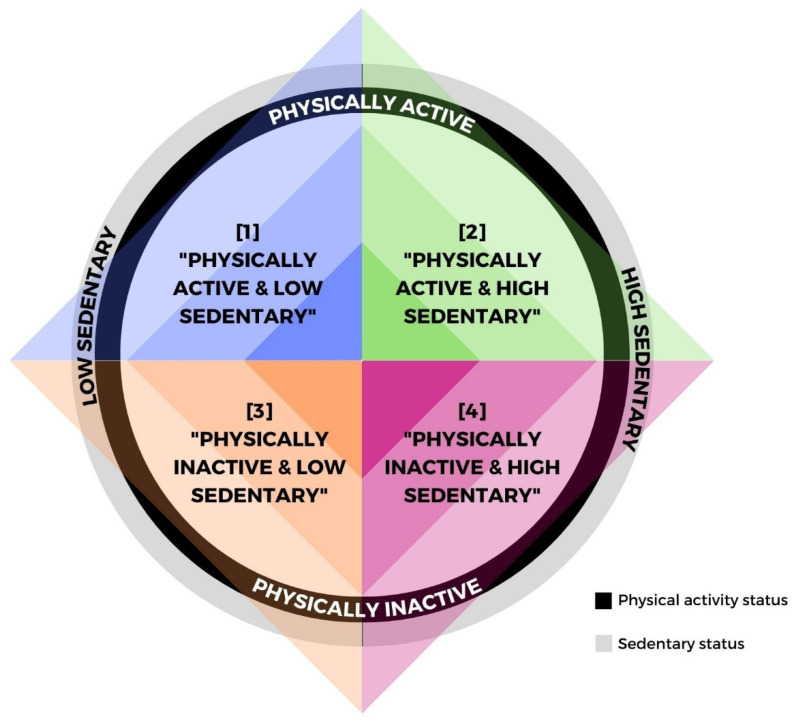
Mutually exclusive behavioural categories. (1) “Physically active and low sedentary”, (2) “Physically active and high sedentary”, (3) “Physically inactive and low sedentary”, (4) “Physically inactive and high sedentary”. Sedentary status was divided into: (1) Low sedentary: quartile 1 according to the number of hours performing sedentary activities during work time and leisure time; (2) High sedentary: quartiles 2, 3 or 4 according to the number of hours performing sedentary activities during work time and leisure time. PA status was divided into: (1) Physically Active: ≥ 150 min of MVPA and ≥ 2 days of strength training per week; (2) Physically Inactive: < 150 min of MVPA and/or < 2 days of strength training per week.

In accordance with Bakrania et al. [13], in this study participants were classified into four patterns based on meeting PA guidelines and sedentary time. As reported by previous studies [14,31], four mutually exclusive behavioural categories were created: (1) “Physically active and low sedentary”, (2) “Physically active and high sedentary”, (3) “Physically inactive and low sedentary” and (4) “Physically inactive and high sedentary” (see in Figure 1).

#### 2.2.5. Covariates

General demographic information (age and sex), type of occupation and number of diseases were collected by means of the questionnaire administered to the participants. Based on the International Standard Classification of Occupations [32], the type of occupation was classified into two groups: work predominantly in facilities or office. The number of diseases included were (1) high cholesterol, (2) high blood pressure, (3) anxiety or depression, (4) diabetes, (5) stroke or other heart disease, (6) myocardial infarction, and (7) others.

### 2.3. Statistical Analysis

Statistical analysis was performed with the SPPS statistical package version 24.0 (IBM Corp., Armonk, NY, USA). Descriptive statistics were expressed as sample size (percentage) and mean ± SD. Those subjects who answered the question regarding how many days they performed MVPA weekly, and subsequently, did not indicate the total time spent on each of those days, were excluded from the analysis. A *p* value of less than 0.05 was considered statistically significant.

#### 2.3.1. Continuous Analysis

To determine differences in HRQoL, different Student’s *t*-tests for two independent samples were performed, including: (1) Workers who complied with aerobic guidelines vs. workers who did not comply with them, (2) Workers who complied with strength guidelines vs. workers who did not comply with them, (3) Workers who complied with aerobic and strength guidelines vs. workers who did not comply with them, and (4) Workers with high sedentary vs. low sedentary status.

#### 2.3.2. Categorical Analysis

Four dichotomous behavioural categories were established, coding as “1” or “0” when subjects met or did not meet the category, respectively. A multiple linear regression model was performed including all behavioural categories as independent variables (excluding the category “physically inactive and high sedentary" considered as reference) and HRQoL as dependent variable. This model was adjusted for covariates (i.e., sex, age, job position and number of diseases).

#### 2.3.3. Minimal Clinically Important Difference (MCID)

The MCID was measured to determine if there was a clinically important difference between the following groups: (1) physically active vs. physically inactive workers, (2) workers with high sedentary vs. low sedentary status and (3) between mutually exclusive behavioural categories. The MCID was calculated following a distribution-based approach using the effect size as proposed by Samsa et al. [33]. To calculate the point equivalent to the MCID, we multiplied the standard deviation of the baseline HRQoL score by 0.2, which was equivalent to a small Cohen’s effect size [34].

## 3. Results

The survey was sent to 1810 workers, and a total of 1055 (58.3%) responded. The final data used in the study excluded 51 participants due to the lack of PA data necessary for their assessment, so a total of 1004 workers were included in the analysis. The sample was composed of 49% workers aged between 36 and 45 years; 76% were male, and the type of occupation was predominantly that of office workers (75%). Forty-nine and thirty-three percent met the WHO aerobic and strength PA guidelines, respectively, resulting in 25% of workers meeting the total guidelines. More detailed information describing the characteristics of the participants is presented in Table 1.

The four behavioural groups were divided as follows: (1) “Physically active and low sedentary” (N = 85; 8.5%), (2) “Physically active and high sedentary” (N = 164; 16.3%), (3) “Physically inactive and low sedentary” (N = 166; 16.5%), and (4) “Physically inactive and high sedentary” (N = 589; 58.7%).

From the analysis regarding meeting aerobic and strength guidelines, those workers who met both types of guidelines, as well as those who met exclusively one of them, had significantly higher levels of HRQoL (*p* < 0.001). Likewise, a high level of sedentary time was significantly associated with a lower HRQoL score (*p* < 0.001) (see more details in Table 2).

**Figure 2:**
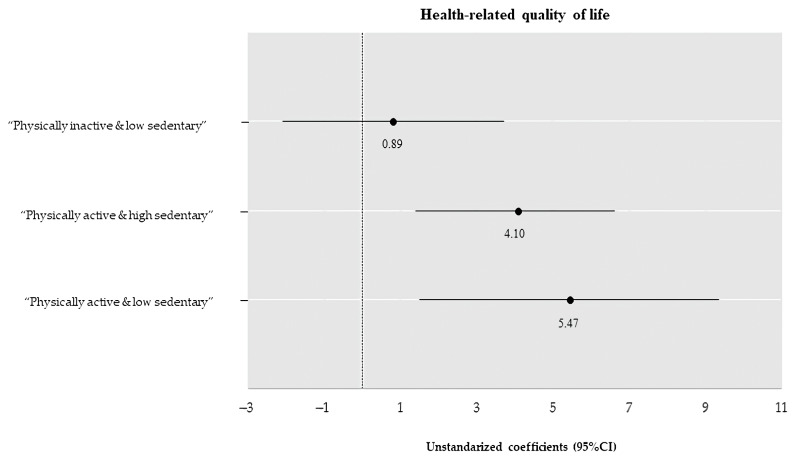
Categorical associations with health-related quality of life (unstandardized regression coefficients (95% CI)). Adjusted linear regression models were fitted for health-related quality of life with the “Physically inactive & high sedentary” category selected as reference group. The models were controlled for: sex, age, type of occupation, and number of diseases.

Compared to “physically inactive and high sedentary” participants, “physically active and low sedentary” and “physically active and high sedentary” individuals had significantly higher levels of HRQoL (B = 5.47; confidence interval [CI] = 1.58, 9.37; *p* = 0.006 and B = 4.10; CI = 1.44, 6.75; *p* = 0.003; respectively) regardless of sex, age, job position and number of diseases. However, no significant association was found for the “physically inactive and low sedentary” group (B = 0.89; CI = −2.18, 3.95; *p* = 0.570) (see in Figure 2).

The MCID for the HRQoL scale score calculated for our study population was 3.22 points. Accordingly, workers who complied with only one type of PA guideline (aerobic or strength) and those who complied with both had a clinically important difference on the HRQoL scale compared to physically inactive workers. Specifically, participants complying with aerobic PA guidelines scored 6.62 points higher on the HRQoL scale compared to those who did not comply with these guidelines. Similar results were obtained in those employees who only met the strength guidelines (4.19 points higher). Participants meeting both types of PA guidelines scored 5.62 points higher on the HRQoL scale compared to those who did not meet both guidelines. Workers with low sedentary time also obtained a clinically relevant difference (3.84 points higher) compared to those with high sedentary time. Regarding the four behavioural categories, “physically active and low sedentary” and “physically active and high sedentary” workers obtained a clinically relevant difference, taking the “physically inactive and high sedentary” behavioural category as reference (differences between groups of 8.52 and 5.15 points, respectively). However, no clinically relevant difference was detected in “physically inactive and low sedentary” workers compared to the reference category (3.15 points).

## 4. Discussion

To our knowledge, this is the first study that quantifies the associations of mutually exclusive behavioural categories with HRQoL outcome in a sample of workers of the Spanish multinational company Grupo Red Eléctrica. As main results, it was found that employees meeting PA guidelines proposed by the WHO (aerobic and strength) and those reporting low sedentary time had a higher HRQoL in this multinational company. Our hypothesis was fulfilled as the analysis of mutually exclusive categories showed that those workers who met the PA guidelines obtained a higher score on the HRQoL scale compared to physically inactive employees, regardless of their sedentary status. Specifically, “physically active and low sedentary” workers obtained the highest score.

Employees spend approximately 60% of their work and waking time engaging in sedentary activities (e.g., working for long periods in front of the computer screen). However, a very small portion (~4%) of the day included the practice of PA [35], something that has been particularly accentuated by the increase in teleworking as a consequence of the current COVID-19 pandemic [36,37]. Our research showed that both workers who independently complied with the aerobic or strength guidelines and those who met both had better HRQoL. The same result was found for those workers who spent less time in sedentary activities. This is in line with the results obtained in the studies carried out by Barranco-Ruiz et al. [9] and Rollo et al. [38], since they showed the existence of a relationship between higher levels of HRQoL in workers who performed more PA and those who were less sedentary, respectively. However, because both compliance with PA guidelines and a sedentary lifestyle are related to HRQoL, and given that they are behaviours that can occur together throughout the day, in our study we analysed four mutually exclusive types of movement behaviour. Findings indicated that employees classified as “physically active and high sedentary” and “physically active and low sedentary” had a positive association with HRQoL, relative to those classified as “physically inactive and high sedentary”. Similar results have been seen in previous studies where these four types of behaviours were related to biomarkers (e.g., triglycerides and insulin) [39], frailty and physical function [14]. In agreement with our results, Loprinzi et al. [39] found that those participants who met PA guidelines were associated with the most beneficial biological marker levels, even in the presence of a high degree of sedentary time. These results are supported by the calculation of the MCID in our sample, as a clinically relevant difference in HRQoL scores was obtained in those workers classified as physically active regardless of their level of sedentary behaviour. Conversely, compared with the reference group, those workers who did not meet the guidelines and had low levels of sedentary time were not significantly associated with better levels of HRQoL and did not have a clinically important difference. The reasons behind these results may be due to the fact that, although having low sedentary time is significantly associated with a higher HRQoL, it is not enough to attenuate the harmful effects of not meeting the PA guidelines, in contrast to the fact that meeting the PA guidelines can attenuate the harmful effects of a sedentary lifestyle.

As expected, the majority of our sample fell into the “physically inactive and high sedentary” behavioural category. Because going from an inactive and sedentary state to meeting minimum PA guidelines, which include aerobic and muscle-strengthening activities, may be too big a step for these people, an intermediate strategy could be to make a gradual and progressive change by tapering off these sedentary times with light-intensity PA [40]. Once the worker has increased their levels of light PA, they can begin to replace it with a combination of aerobic and strength activities that will allow them to meet the recommended PA levels. Future research should demonstrate whether the practice of light PA can increase workers’ HRQoL.

In line with our results, if companies have to prioritise resources, the main efforts should be aimed towards facilitating the inclusion of PA in workers’ day-to-day life instead of reducing the sedentary time, either inside or outside the workplace. In this sense, there is evidence that highlights the beneficial effects of increasing employees’ PA levels, both for the workers themselves [41] and the company, as it results in reduced absenteeism [42], more engaged employees [43], and better performance [44].

### Limitations

Some limitations of this study should be acknowledged. Firstly, given the observational nature of the study, it was not possible to know the direction of the associations between the variables analysed. However, all of the questions included were based on questionnaires that were validated for that purpose. Furthermore, this is the first study that analyses the association between the four categories of PA behaviour and sedentary time with the HRQoL of workers of a Spanish multinational company within an IBEX-35 company. IBEX 35 is the main benchmark stock market index of the Spanish stock market, consisting of the 35 most liquid companies listed on the Spanish Stock Exchange Interconnection System on the four Spanish stock exchanges (Madrid, Barcelona, Bilbao and Valencia). Companies in this group tend to have a more complicated internal organisational and functional structure, which can make it difficult to access their data for research purposes. However, the data obtained on this type of large-scale enterprise can be used as a reference for other, small and medium-sized companies. Secondly, having been carried out in Grupo Red Eléctrica company, another possible limitation is that the results obtained cannot be extrapolated to the general population. Nevertheless, the results obtained in the study have practical application since they allow companies to orient their efforts in the right direction to provide intervention strategies that improve the HRQoL of their workers. Finally, there is no gold standard for calculating MCID, and the results are generally very heterogeneous. Therefore, the results derived from the comparison with the calculated MCID should be interpreted with caution. Future workplace intervention studies should investigate whether reducing sedentary behavior and especially promoting compliance with PA guidelines result in clinically relevant differences.

## 5. Conclusions

We found that both sedentary time and meeting the PA guidelines (aerobic and strength) proposed by the WHO were significantly related to HRQoL in the employees of this multinational company. Meeting the PA guidelines was associated with a higher HRQoL score regardless of the level of sedentarism. Our findings suggest that, whenever possible, the company should promote strategies to decrease sedentary time and increase PA practice. However, according to our results, if the company had to prioritise resources, it seems that increasing the time allocated to meeting WHO PA guidelines may have greater implications for employees’ HRQOL. Further clarification is needed to reaffirm our results through experimental research studies.

## Figures and Tables

**Table 1:** Participant characteristics.

Characteristics	Total Sample	“Physically Active & Low Sedentary”	“Physically Active & High Sedentary”	“Physically Inactive & Low Sedentary”	“Physically Inactive & High Sedentary”
	N = 1004	N = 85; 8.5%	N = 164; 16.3%	N = 166; 16.5%	N = 589; 58.7%
Age (years)					
Less than 25 years	7 (0.7)	0 (0.0)	3 (1.8)	0 (0.0)	4 (0.7)
From 26 to 35 years	150 (14.9)	20 (23.5)	29 (17.7)	26 (15.7)	75 (12.7)
From 36 to 45 years	488 (48.6)	44 (51.8)	80 (48.8)	80 (48.2)	284 (48.2)
From 46 to 55 years	207 (20.6)	13 (15.3)	29 (17.7)	29 (17.5)	136 (23.1)
More than 55 years	152 (15.1)	8 (9.4)	23 (14)	31 (18.7)	90 (15.3)
Sex					
Male	766 (76.3)	82 (96.5)	128 (78.0)	142 (85.5)	414 (70.3)
Female	238 (23.7)	3 (3.5)	36 (22.0)	24 (14.5)	175 (29.7)
Type of occupation					
Facilities workers	252 (25.1)	61 (71.8)	20 (12.2)	112 (67.5)	59 (10.0)
Office workers	752 (74.9)	24 (28.2)	144 (87.8)	54 (32.5)	530 (90.0)
Number of diseases					
0	430 (42.8)	50 (58.8)	76 (46.3)	80 (48.2)	224 (38.0)
1	370 (36.9)	22 (25.9)	64 (39.0)	59 (35.5)	225 (38.2)
2	148 (14.7)	12 (14.1)	19 (11.6)	17 (10.2)	100 (17.0)
3	45 (4.5)	1 (1.2)	4 (2.4)	7 (4.2)	33 (5.6)
4	10 (1.0)	0 (0.0)	1 (0.6)	3 (1.8)	6 (1.0)
5	1 (0.1)	0 (0.0)	0 (0.0)	0 (0.0)	1 (0.2)
≥6	0 (0.0)	0 (0.0)	0 (0.0)	0 (0.0)	0 (0.0)
Meet WHO aerobic guidelines					
Yes	494 (49.2)	85 (100.0)	164 (100.0)	69 (41.6)	176 (29.9)
No	510 (50.8)	0 (0.0)	0 (0.0)	97 (58.4)	413 (70.1)
Meet WHO strength guidelines					
Yes	327 (32.6)	85 (100.0)	164 (100.0)	16 (9.6)	62 (10.5)
No	677 (67.4)	0 (0.0)	0 (0.0)	150 (90.4)	527 (89.5)
Meet WHO aerobic & strength guidelines					
Yes	249 (24.8)	85 (100.0)	164 (100.0)	0 (0.0)	0 (0.0)
No	755 (75.2)	0 (0.0)	0 (0.0)	166 (100.0)	589 (100.0)
Sedentary time					
High	753 (75.0)	0 (0.0)	164 (100.0)	0 (0.0)	589 (100.0)
Low	251 (25.0)	85 (100.0)	0 (0.0)	166 (100.0)	0 (0.0)
HRQoL score	77.17 ± 16.11	83.60 ± 10.39	80.25 ± 15.16	78.23 ± 14.32	75.08 ± 17.12

N (proportion (%)); mean ± standard deviation; HRQoL: health-related quality of life; WHO: World Health Organization.

**Table 2:** HRQoL values between active workers vs. inactive workers and low sedentary vs. high sedentary workers.

	Active	Inactive	Low Sedentary	High Sedentary	*p*-Value
**MVPA guidelines**	N = 494 80.53 ± 14.44	N = 510 73.91 ± 16.97	_	_	<0.001 *
**Strength guidelines**	N = 327 79.99 ± 15.09	N = 677 75.8 ± 16.43	_	_	<0.001 *
**MVPA + Strength guidelines**	N = 249 81.39 ± 13.79	N = 755 75.77 ± 16.58	_	_	<0.001 *
**Sedentary status**	_	_	N = 251 80.05 ± 13.34	N = 753 76.21 ± 16.84	0.001 *

Values are expressed as mean ± standard deviation; * *p* < 0.05; MVPA: moderate-to-vigorous physical activity.

## Data Availability

All data used for this analysis can be acquired at any time by contacting the corresponding author.
